# Review of the evolution of insecticide resistance in main malaria vectors in Cameroon from 1990 to 2017

**DOI:** 10.1186/s13071-017-2417-9

**Published:** 2017-10-10

**Authors:** Christophe Antonio-Nkondjio, N. Sonhafouo-Chiana, C. S. Ngadjeu, P. Doumbe-Belisse, A. Talipouo, L. Djamouko-Djonkam, E. Kopya, R. Bamou, P. Awono-Ambene, Charles S. Wondji

**Affiliations:** 1 Laboratoire de Recherche sur le Paludisme, Organisation de Coordination pour la lutte Contre les Endémies en Afrique Centrale (OCEAC), Yaoundé, Cameroon; 2 Faculty of Health Sciences, University of Buea, Buea, Cameroon; 3 Faculty of Sciences, University of Yaoundé I, Yaoundé, Cameroon; 4 Faculty of Sciences, University of Dschang, Dschang, Cameroon; 5 Vector Group, Liverpool School of Tropical Medicine, Pembroke Place, Liverpool, UK

**Keywords:** Insecticide resistance, *An. gambiae* (*s.l.*), *An. funestus*, Vector control, Malaria, Cameroon

## Abstract

**Background:**

Malaria remains a major public health threat in Cameroon and disease prevention is facing strong challenges due to the rapid expansion of insecticide resistance in vector populations. The present review presents an overview of published data on insecticide resistance in the main malaria vectors in Cameroon to assist in the elaboration of future and sustainable resistance management strategies.

**Methods:**

A systematic search on mosquito susceptibility to insecticides and insecticide resistance in malaria vectors in Cameroon was conducted using online bibliographic databases including PubMed, Google and Google Scholar. From each peer-reviewed paper, information on the year of the study, mosquito species, susceptibility levels, location, insecticides, data source and resistance mechanisms were extracted and inserted in a Microsoft Excel datasheet. The data collected were then analysed for assessing insecticide resistance evolution.

**Results:**

Thirty-three scientific publications were selected for the analysis. The rapid evolution of insecticide resistance across the country was reported from 2000 onward. Insecticide resistance was highly prevalent in both *An. gambiae* (*s*.*l*.) and *An. funestus*. DDT, permethrin, deltamethrin and bendiocarb appeared as the most affected compounds by resistance. From 2000 to 2017 a steady increase in the prevalence of *kdr* allele frequency was noted in almost all sites in *An. gambiae* (*s*.*l*.), with the L1014F *kdr* allele being the most prevalent. Several detoxification genes (particularly P450 monooxygenase) were associated with DDT, pyrethroids and bendiocarb resistance. In *An. funestus*, resistance to DDT and pyrethroids was mainly attributed to the 119F-GSTe2 metabolic resistance marker and over-expression of P450 genes whereas the 296S-RDL mutation was detected in dieldrin-resistant *An. funestus*.

**Conclusions:**

The review provides an update of insecticide resistance status in malaria vector populations in Cameroon and stresses the need for further actions to reinforce malaria control strategies in the coming years.

## Background

The rapid expansion of insecticide resistance in malaria vectors resulting from the frequent use of similar insecticide families for public health and agriculture could end up jeopardizing control efforts. The situation requires further attention to improve strategies toward malaria elimination in Africa. Many countries have reported resistance to pyrethroids, the main compound used for bednets impregnation [1]. Apart of pyrethroids, the remaining insecticide families (organochlorines, carbamates, organophosphates) are also affected at different levels [2]. All major vectors in Africa, i.e. *An. gambiae* (*s*.*l*.), *An. coluzzii*, *An. arabiensis* and *An. funestus*, have developed resistance to at least one compound [2–5]. Monitoring the evolution of insecticide resistance in vector populations is becoming urgent to preserve the efficacy of insecticide tools currently used for vector control (LLINs, IRS) and to guide future vector control interventions [6]. In Cameroon, despite increase efforts to control malaria the disease remains largely prevalent across the country [7]. Since 2000, the Ministry of Health has implemented three important programmes of mass distribution of impregnated bed nets to the population [7–9]. These efforts have increased ownership and use of impregnated bed nets [9, 10] and brought about a decrease in malaria-induced morbidity and mortality [1, 10]. These efforts are hindered by the rapid expansion of insecticide resistance across the country with several species being affected [11–14]. Insecticide resistance has been reported to be highly prevalent in urban and rural agricultural settings [12, 15, 16]. Since the 1990s, monitoring of mosquito population susceptibility or characterisation of resistance mechanisms have been the subject of many studies conducted across the country. However, the data from these studies have never been comprehensively analysed together. Here, we comprehensively analyse data from 33 publications on mosquito susceptibility to insecticides and resistance mechanisms in vector populations across Cameroon to (i) highlight challenges awaiting malaria vector control efforts; (ii) develop an understanding of the evolution of insecticide resistance in these vector populations; and (iii) use the meta-analysis data to guide future vector control strategies to be implemented across Cameroon.

## Methods

### Search method

Data on insecticide resistance in malaria vectors in Cameroon were extracted from published reports. Online bibliographic databases including PubMed, Google and Google Scholar were used to search for the information. Terms used to guide these searches included “insecticide resistance”, “*Anopheles*”, “Cameroon”, “susceptibility”. The search period included 1987 to 2017 because studies on malaria vectors resumed at this time. All scientific publications reporting information on vector bionomic or malaria transmission by mosquitoes were also screened.

### Data extraction and consideration

Published works were included in the study if they reported information on insecticide resistance or mosquito susceptibility to insecticides. Excluded from the review were papers reporting susceptibility tests done with laboratory colonies, experimental huts trials, bioassays conducted with anopheline larvae, modelling studies, studies assessing bednet bio-efficacy with laboratory colonies and unpublished data.

From each selected published study, the following data were extracted and inserted in a Microsoft Excel datasheet: the year of the study, method for assessing mosquito susceptibility, mosquito species studied, location, site characteristics, insecticides tested (pyrethroids, organochlorines, carbamates, organophosphates), data source, resistance mechanisms and susceptibility level. The data were then analysed to retrieve information on mosquito susceptibility or insecticide resistance. According to the World Health Organization criteria [17], a resistant mosquito population displays a mortality rate < 90%, a susceptible population has a mortality rate > 97%, whereas populations with mortality between 90 and 97% needed confirmation.

## Results

### Literature search

A first selection round was undertaken with a total of 46 studies preselected of which 13 were excluded because they did not met inclusion criteria. A total of 33 published studies written in French or English were selected for review. These publications reported studies undertaken between 1990 and 2017. Table 1 provides a summary of the information extracted from these papers. Most of the studies reported on *An. gambiae* (*s*.*l*.) population susceptibility to DDT, permethrin and deltamethrin, while a few reported tests with carbamates and organophosphates. Studies on insecticide resistance assessed *kdr* allele presence and distribution and metabolic base mechanisms.

**Table 1:** Summary of data collected in peer-review papers selected for the study

Reference	Study year	Species	Insecticide	Location	Topics covered
[42]	2006	*An. gambiae* (*s*.*l*.)	DDT, perm, lamb, delta, feni, prop	1	Susceptibility & *kdr* resistance
[62]	2011	*An. gambiae* (*s*.*l*.)	DDT, perm, delta	2, 3	Susceptibility
[26]	1998	Anopheline	cyflu	4	Tool evaluation
[58]	2013	*An. gambiae* (*s*.*l*.)	perm	3	Susceptibility
[13]	2010	*An. gambiae* (*s*.*l*.)	perm, delta	2, 3	Susceptibility & *kdr* resistance
[52]	2010	*An. gambiae* (*s*.*l*.)	bend	3	Susceptibility & metabolic resistance
[34]	2006	*An. gambiae* (*s*.*l*.)	carbo, delta, perm, DDT	5, 6, 7	Susceptibility & *kdr* resistance
[63]	2011	*An. gambiae* (*s*.*l*.)	DDT, perm, delta, lamb, bend, mala, cyflu	8	Susceptibility
[44]	1998	*An. gambiae sl*	DDT, perm, delta	3	Susceptibility & *kdr* resistance
[12]	2005	*An. gambiae* (*s*.*l*.)	lamb, mala, perm, chlorpy, prop, DDT, delta	9, 10, 11	Susceptibility & *kdr* resistance
[43]	2000	*An. gambiae* (*s*.*l*.)	DDT, perm, delta	2, 3, 12, 9, 13, 11, 14, 15, 16	Susceptibility & *kdr* resistance
[28]	2003–2004	*An. gambiae* (*s*.*l*.)	DDT, perm, delta	17, 2, 18	Susceptibility & *kdr* resistance
[64]	2006	*An. gambiae* (*s*.*l*.)		2, 12, 11, 15	Metabolic resistance
[65]	2006	*An. gambiae* (*s*.*l*.)		19, 20, 5, 2, 21, 18, 23	*kdr* resistance
[66]		*An. gambiae* (*s*.*l*.)		3	Susceptibility
[11]	2013	*An. gambiae* (*s*.*l*.)	DDT, perm, delta	24, 25, 11	Susceptibility
[24]	1990	Anopheline	delta & ITNs	26	Susceptibility
[23]	1990	Anopheline	DEET	26	Tool evaluation
[25]	1995	Anopheline	coils and mats	27	Tool evaluation
[25]	1993–1994	Anopheline	coils and mats	27	Tool evaluation
[14]	2012	*An. funestus*	etof, feni, diel, mala, bend, DDT, lamb, delta, perm	1	Susceptibility & resistance
[51]	2006	*An. gambiae* (*s*.*l*.)	pyrethroids	11	Metabolic resistance
[29]	2002	*An. gambiae* (*s*.*l*.)	DDT, perm, delta, lamb, bend, mala	29, 30, 1, 31, 23, 3, 41, 42, 32, 33, 34, 17, 19 35, 36, 5, 37	Susceptibility & *kdr* resistance
[67]	2013	*An. gambiae* (*s*.*l*.)	DDT, perm, delta	2	Susceptibility
[68]	2015	*An. gambiae* (*s*.*l*.)	DDT, perm, delta	2	Susceptibility
[16]	2001	*An. gambiae* (*s*.*l*.)	DDT, diel, lamb, delta, perm	2, 39, 3, 40	Susceptibility & *kdr* resistance
[69]	2006	*An. gambiae* (*s*.*l*.)		3, 23, 2, 39, 18, 21, 2, 19, 5, 7, 40, 11, 20, 43, 38, 31	*kdr* resistance
[35]	2008–2009	*An. gambiae* (*s*.*l*.)	DDT, perm, delta	2, 23, 18, 3, 38, 11	Susceptibility & *kdr* resistance
[70]	2006	*An. gambiae* (*s*.*l*.)	DDT, perm, delta	35, 23	*kdr* resistance
[71]	2005	*An. gambiae* (*s*.*l*.)		21, 40, 23	*kdr* resistance
[50]	2012	*An. gambiae* (*s*.*l*.)	DDT	3	Metabolic resistance
[3]	2006	*An. funestus*	dieldrin	28	Susceptibility & resistance
[30]	2011–2015	*An. gambiae* (*s*.*l*.)	delta	9, 11, 44	Susceptibility & *kdr* resistance

*Abbreviations*: *Perm* permethrin, *delta* deltamethrin, *lamb* lambda-cyhalothrin, *bend* bendiocarb, *mala* malathion, *diel* dieldrin, *prop* propoxur, *etof* etofenprox, *carbo* carbosulfan, *cyflu* cyfluthrin

*Locations*: Gounougou (1), Douala (2), Yaoundé (3), Mbandjock (4), Tiko (5), Limbe (6), Idenau (7), Niété (8), Garoua (9), Gashiga (10), Pitoa (11), Mbalmayo (12), Boklé (13), Sekandi (14), Simatou (15), Maouda (16), Foumbot (17), Campo (18), Loum (19), Magba (20), Kribi (21), Akonolinga (22), Bertoua (23), Manoka (24), Youpwe (25), Mbebe (26), Nsimalen (27), Lagdo (28), Kousseri (29), Maga (30), Ngaoundéré (31), Mengong (32), Djoum (33), Ndop (34), Santchou (35), Bonassama (36), Nkongsamba (37), Makoutchietoum (38), Ipono (39), Mangoum (40), Soa (41), Dabadi (42), Bonamikengué (43), Mayo Oulo (44)

### Distribution of members of the *An. gambiae* complex and *An. funestus*

**Fig. 1:**
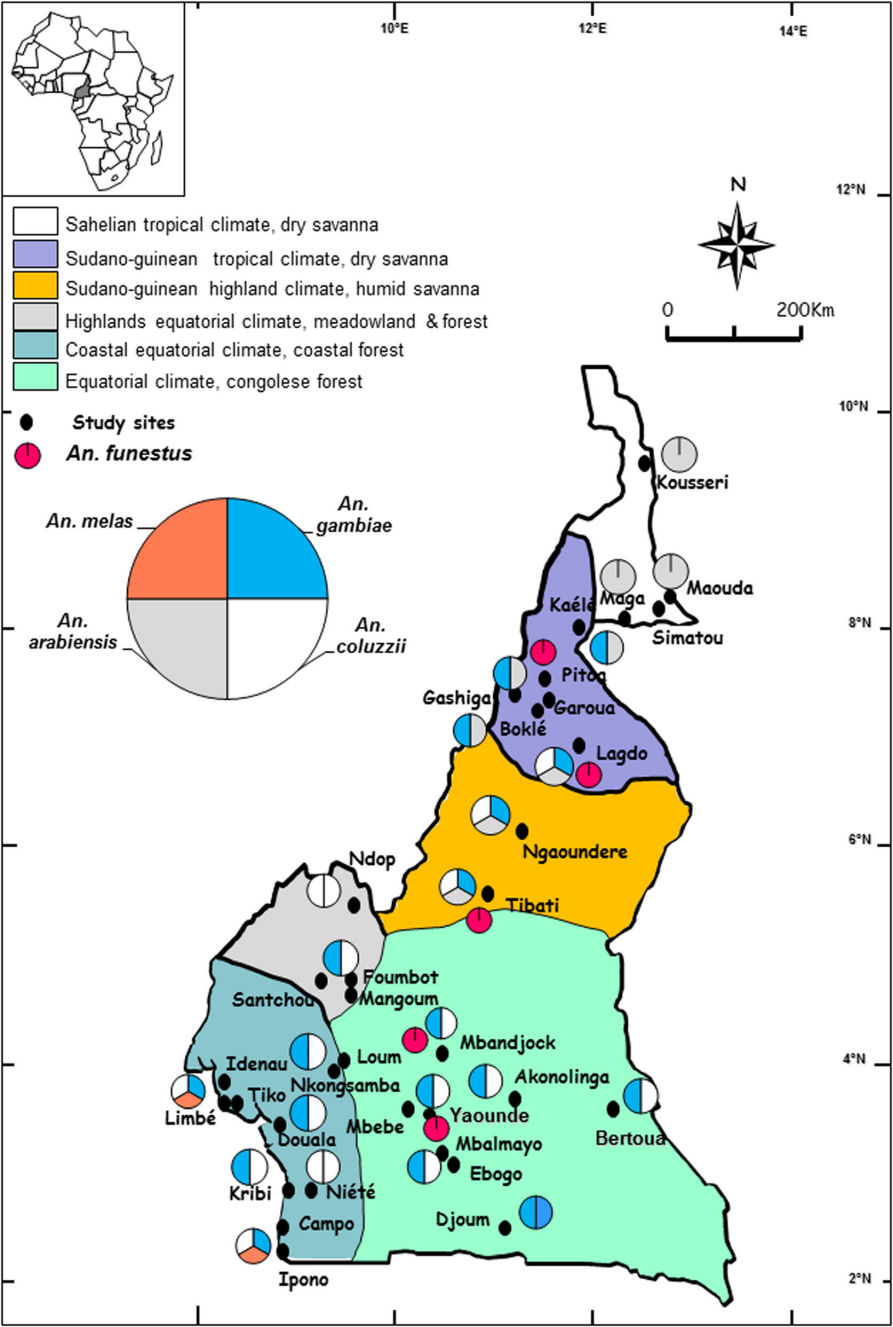
A map of Cameroon showing the distribution of study sites and members of the *An. gambiae* complex and *An. funestus*

Members of the *An. gambiae* complex largely distributed in south Cameroon, are *An. gambiae* and *An. coluzzii*. However, in most urban settings *An. coluzzii* densities are far more important than those of *An. gambiae* which is more prevalent in periurban and rural settings [18, 19]. *Anopheles melas* is found near the Atlantic coast [20]. In the northern part of the country, *An. arabiensis* and *An. gambiae* are the main species of the complex with however *An. arabiensis* always dominating (Fig. 1) [18]. *Anopheles funestus* has a large distribution and is present across the country [21, 22].

### Studies conducted between 1990 and 1998

Before the official introduction of treated bednets for malaria vector control in Cameroon, several trials were implemented between 1990 to 1998 to assess the efficacy of various insecticide tools such as treated nets, insecticide spray or coils against mosquito populations [23–26]. A household survey conducted in the city of Douala during that period indicated that 48% of households were using non-impregnated bednets, 39.5% insecticide spray and 36.7% coils [27]. In the absence of field monitoring data on mosquito susceptibility level to insecticides, the result of these trials could provide indications on the level of susceptibility of mosquito populations at that period. A reduction of 48–83% of the biting rate of vectors such as *An. gambiae* (*s*.*l*.), *An. funestus*, *An. nili* and *An. moucheti* was reported using these tools. In studies using treated nets, a reduction of 74–90% of the entomological inoculation rate was also reported (Table 2).

**Table 2:** Reported impact of some field trials conducted in the early 1990s before mass distribution of treated bed nets to the population in rural forest region of south Cameroon

Study site [source]	Tools tested	Main species	Reduction in			Year of the trial
			HBR (%)	EIR (%)	Malaria prevalence (%)	
Mbébé [24]	ITN (deltamethrin treated nets: 25 mg/m^2^)	*An. nili*; *An. gambiae* (*s*.*l*.); *An funestus*	62	78	75	1990
Kumba [72]	ITN (deltamethrin treated nets: 25 mg/m^2^)	*An. gambiae*			30	1992
Ebogo [36]	ITN (lambdacyhalothrin: 15 mg/m^2^)	*An. moucheti*	74	90	40	1992
Mbébé [23]	DEET (50% diethyl-toluamide)	*An. nili*; *An. gambiae* (*s*.*l*.); *An funestus*	48			1992
Nsimalen [25]	Coils (0.15% *w*/w of esbiothrin)	*An. moucheti*; *An. gambiae* (*s*.*l*.); *Mansonia* sp.	78			1993–1994
	Mats (d-allethrin; S biothrin and diethyl-toluamide)	*An. moucheti*; *An. gambiae* (*s*.*l*.); *Mansonia sp.*	83.3			
Mbandjock [26]	ITN (cyfluthrin-treated nets EW050)	*An. gambiae* (*s*.*l*.); *An. funestus*; *An. nili*; *An. moucheti*	0	74		1997–1998

*Abbreviations*: *HBR* human biting rate, *EIR* entomological inoculation rate, *ITN* insecticide-treated nets

### Evolution of mosquito susceptibility to insecticides after 1998

**Fig. 2:**
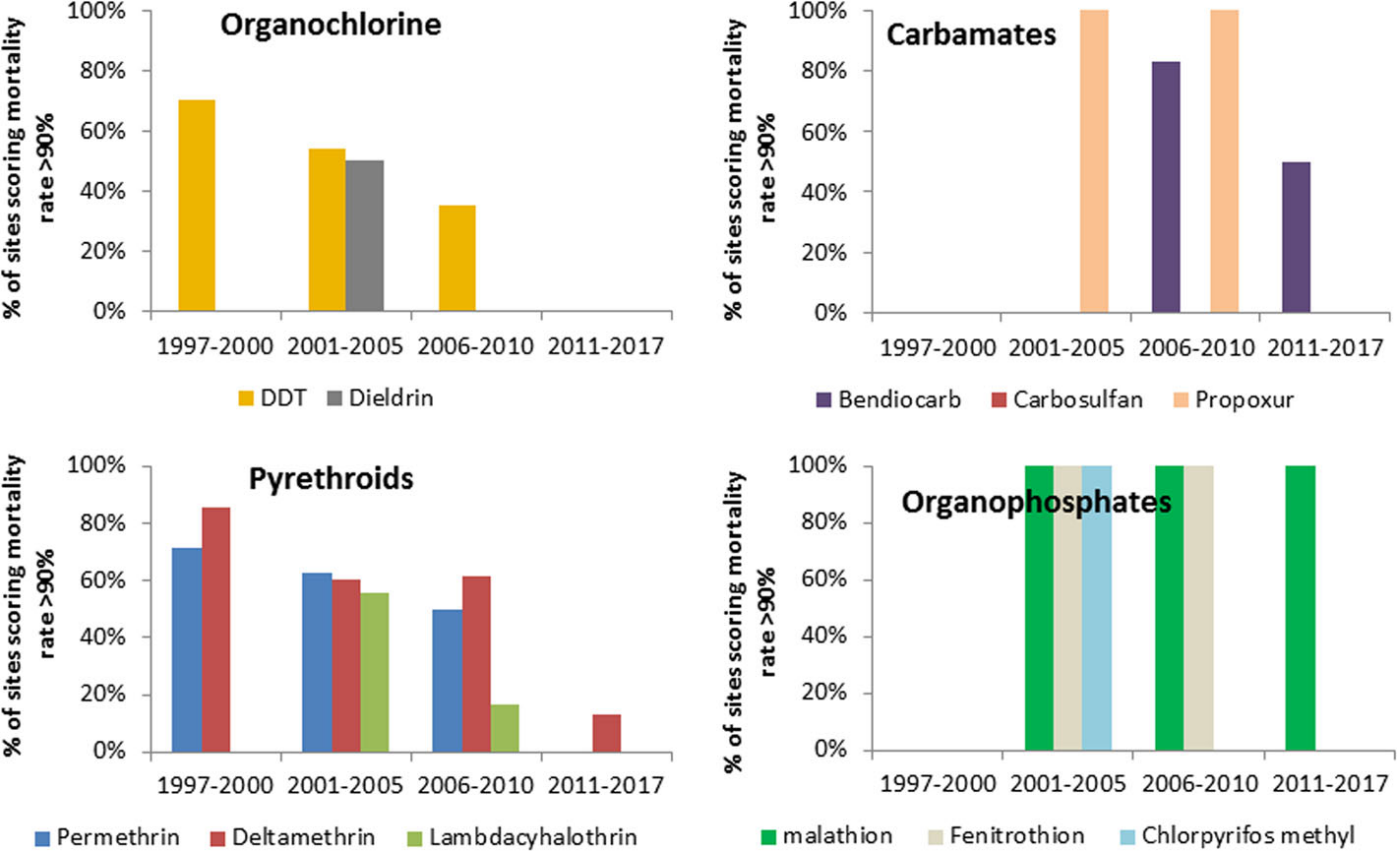
Proportion of sites scoring high susceptibility (mortality rate > 90%) of *An. gambiae* populations to organochlorines, pyrethroids, carbamates and organophosphates in Cameroon between 1998 and 2017

From 1998 onward WHO tube tests were frequently used across the country to monitor mosquito susceptibility to insecticides. Four compounds were regularly monitored: DDT, permethrin, deltamethrin and lambdacyhalothrin. Screening of mosquito population susceptibility was undertaken in 44 locations across the country. The proportion of sites reporting high susceptibility of *An. gambiae* (*s*.*l*.) to insecticides (mortality rate > 90%) decreased gradually. In the period between 1998 and 2000, over 85% of sites (6/7) reported a high susceptibility level (mortality rate > 90%) of *An. gambiae* (*s*.*l*.) to deltamethrin and a few cases of resistance were reported for DDT (3 out of 10 sites) and permethrin (2 out of 7 sites) at Yaoundé, Douala and Mbalmayo where *An. gambiae* and *An. coluzzii* are the most prevalent species (Fig. 2). Resistance to Dieldrin was detected in all sites. From 2001 to 2005, about 53% of sites (14/26 sites) were scoring high susceptibility (mortality rate > 90%) to DDT. For permethrin, deltamethrin and lambdacyhalothrin, the proportion of sites scoring high mortality to these compounds were 60.5% (*n* = 27), 55% (*n* = 9) and 63% (*n* = 27), respectively. The period between 2006 and 2010 saw a significant increase in the level of resistance of *An. gambiae* (*s*.*l*.) populations to DDT and pyrethroids. High susceptibility to DDT was recorded only in 35% of sites (7 out of 20) whereas, 50% (11/22), 62% (13/21) and 17% (1/6) of the sites were reporting high susceptibility to permethrin, deltamethrin and lambdacyhalothrin, respectively (Fig. 2). An increase in the level of resistance of *An. gambiae* (*s*.*l*.) to carbamates (carbosulfan and bendiocarb) was also recorded. From 2011 to 2017, almost all sites across the country reported high resistance levels of *An. gambiae* (*s*.*l*.) populations to DDT, permethrin and deltamethrin. Resistance to the carbamate bendiocarb was also reported in a few sites. Also, high susceptibility to organophosphates was continually observed at all sites (Fig. 2).

**Fig. 3:**
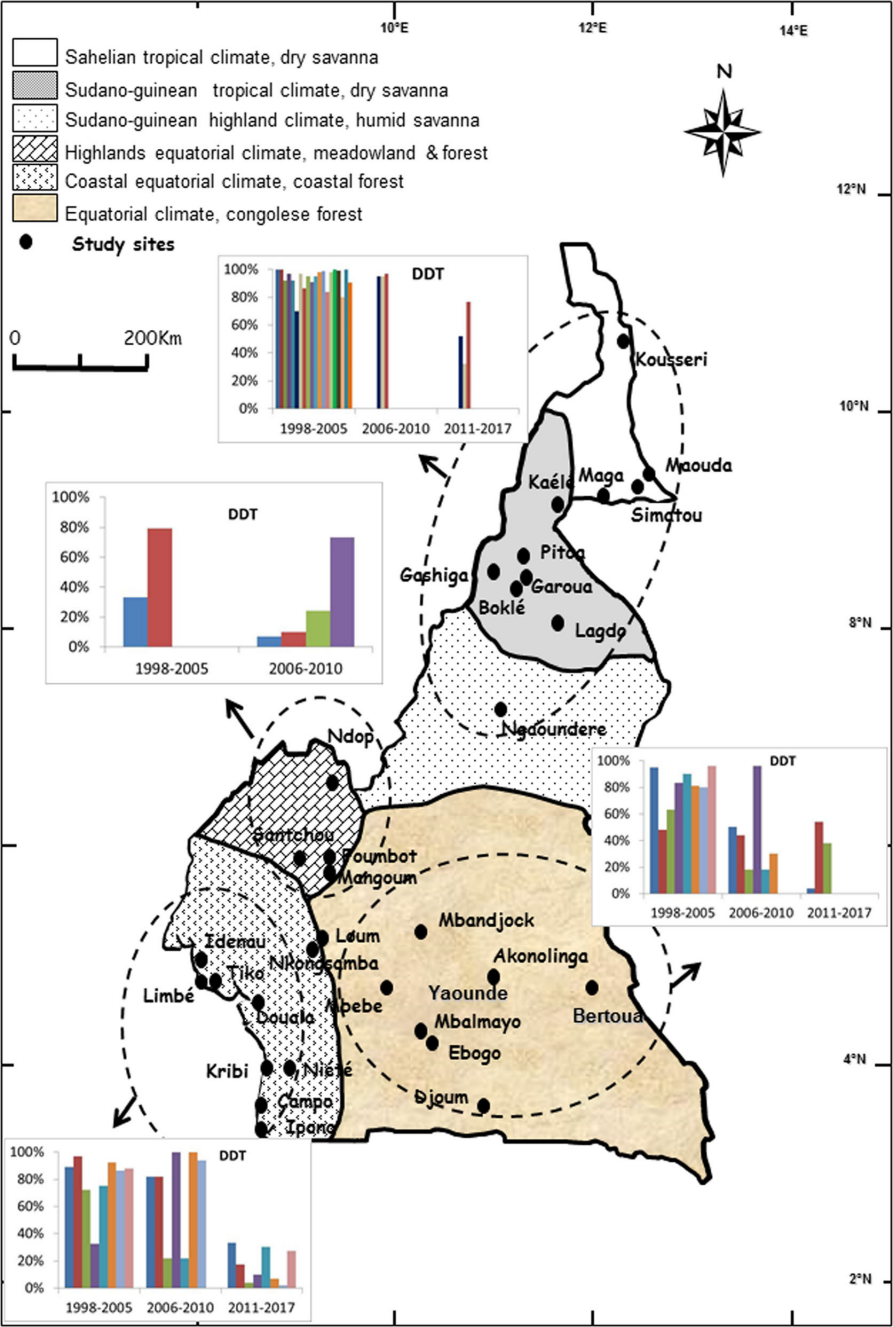
Evolution of *An. gambiae* (*s.l.*) populations mortality rate to DDT in different sites across Cameroon from 1998 to 2017

**Fig. 4:**
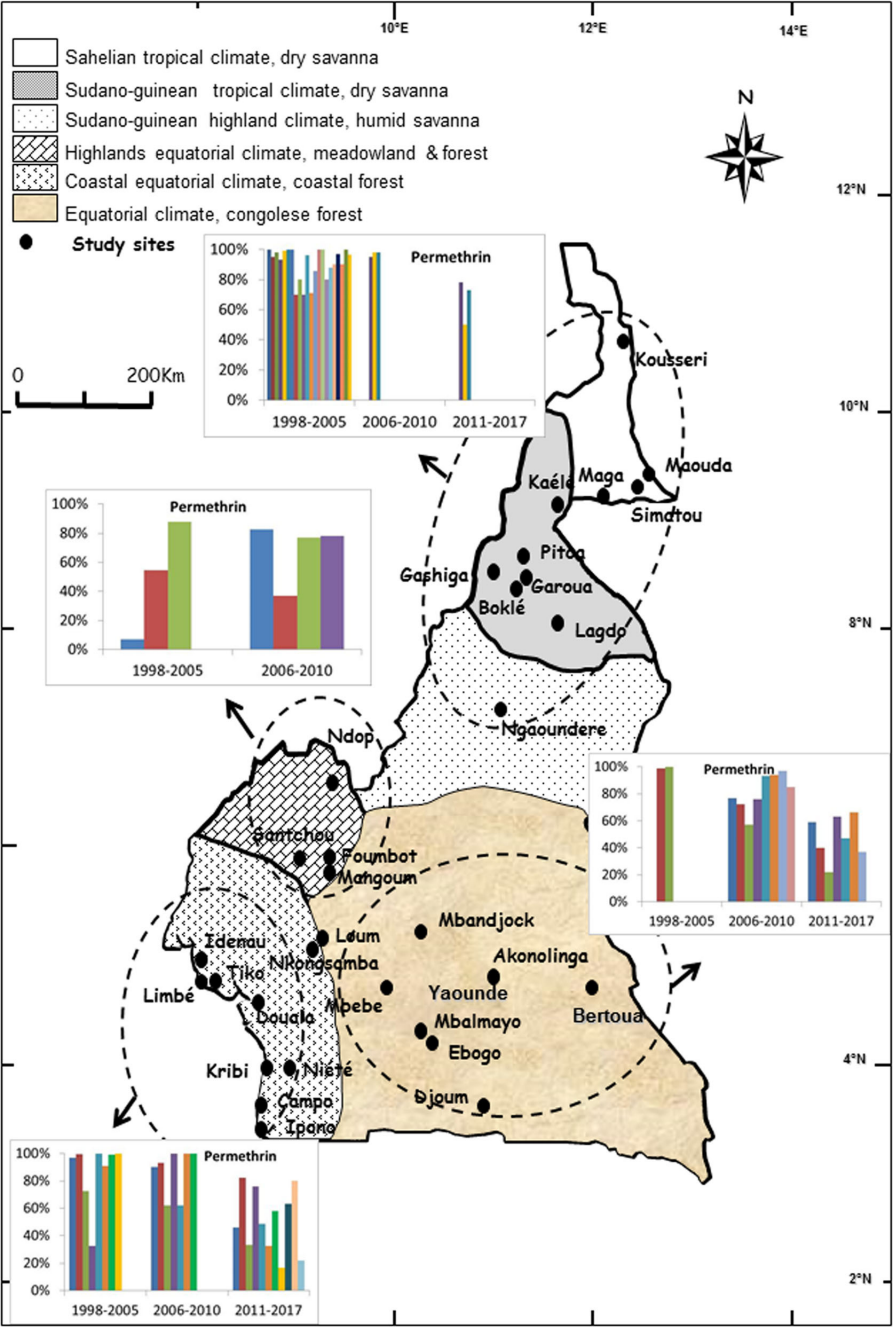
Evolution of *An. gambiae* (*s.l.*) populations mortality rate to Permethrin in different sites across Cameroon from 1998 to 2017

**Fig. 5:**
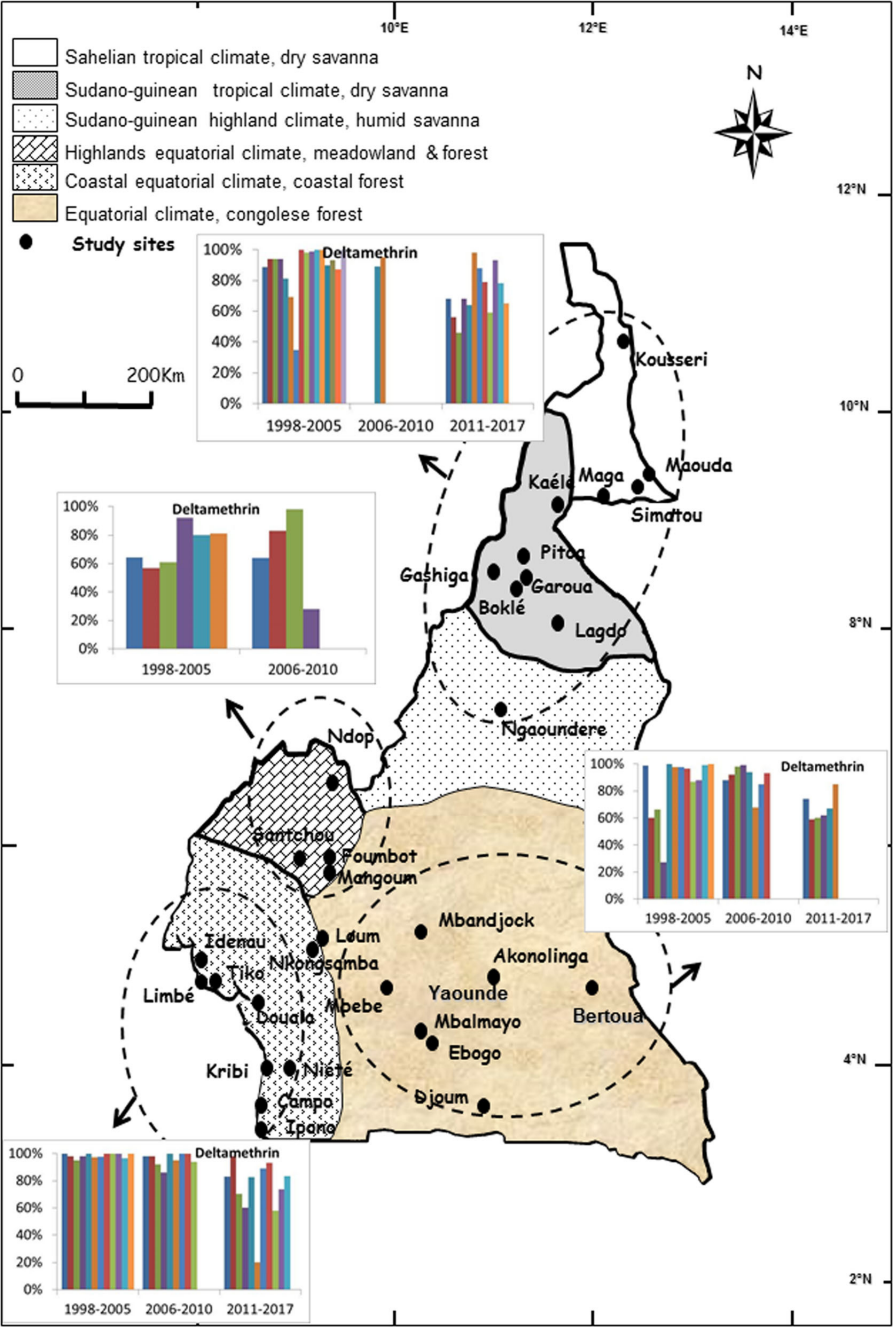
Evolution of *An. gambiae* (*s.l.*) populations mortality rate to Deltamethrin in different sites across Cameroon from 1998 to 2017

The data on mosquito susceptibility were then reorganized according to the main geographical and ecological divisions of the country and analysed to assess the pattern of evolution of *An. gambiae* (*s*.*l*.) population resistance to DDT, permethrin and deltamethrin. This analysis permitted the assessment of variation of mosquito population susceptibility levels at different periods. Although resistance to both DDT and pyrethroids emerged rather quickly in the western highlands area where intensive agriculture is practised by the population [16, 28], a gradual increase in the *An. gambiae* (*s*.*l*.) population tolerance to DDT, permethrin and deltamethrin was recorded in the remaining geographical settings suggesting increased insecticide selection occurring across all geographical areas (Figs. 3, 4 and 5).

The screening of *An. funestus* populations started in 2006 but most studies on this species were conducted between 2011 and 2015 in Gounougou, where a high level of insecticide resistance for organochlorine, pyrethroids and carbamates was recorded [14] (Table 3).

**Table 3:** Evolution of susceptibility to insecticides and resistance mechanisms in *Anopheles funestus* populations in Cameroon between 1997 and 2017

Insecticide family	Insecticide	No. of sites with high susceptibility to insecticide (mortality > 90%)/ total number of sites (range)			
		1997–2000	2000–2005	2006–2010	2011–2017
Organochlorines	DDT	–	–	–	0/2 (32–52%)
	Dieldrine	–	–	1/1 (20%)	0/1 (26%)
Organophosphate	Malathion	–	–	–	2/2 (100%)
	Fenitrothion	–	–	–	0/1 (78%)
Carbamates	Bendiocarb	–	–	–	0/2 (86–88%)
Pyrethroids	Permethrin	–	–	–	0/2 (50–78%)
	Deltamethrin	–	–	–	0/2 (64–68%)
	Lambda-cyhalothrin	–	–	–	0/1 (48%)
	Etofenprox	–	–	–	0/1 (68%)

### Insecticide resistance mechanisms characterisation

From 1998 to 2017, a gradual increase in the *kdr* allele frequencies was recorded in almost all settings. Both *kdr* alleles L1014S from East and L1014F from West Africa were recorded: the West African *kdr* allele (L1014F) was the most prevalent (Table 4). Although *An. arabiensis* was found to rarely possess the *kdr* allele [29] between 2000 and 2010, more recent investigations indicate a high prevalence of the *kdr* allele L1014F in this species [30, 31].

**Table 4:** Evolution of the *kdr* allele frequency in *Anopheles gambiae* (*s.l.*) populations in different ecological settings across Cameroon between 1997 and 2017

Sites		*Kdr* allele frequency (%)			
	*Kdr* alleles	1997–2000	2001–2005	2006–2010	2011–2017
Urban sites (Forest areas)^a^	L1014F	0	0–75.0	6.8–98.0	33.0–98.0
	L1014S	0	8.3	0–21.0	–
Rural sites (Forest areas)^b^	L1014F	–	4.0–53.4	5.0–62.7	69.7
	L1014S	0	0	0–40.0	–
Urban sites (Sahelian, dry and humid savanna areas)^c^	L1014F	0	–	18.7	15.0–97.0
	L1014S	0	–	3.1	–
Rural sites (Sahelian, dry and humid savanna areas)^d^	L1014F	0	2.5	0–62.0	0–100
	L1014S	0	–	0–18.0	2.0
Rural sites (grassfields Highland area)^e^	L1014F	0	37.0–84.9	38.0–84.0	–
	L1014S	0	4.8–14.5	0–24.0	–

^a^Urban sites (forest and humid savanna area) include Douala, Yaoundé, Bertoua, Kribi, Limbe, Mbalmayo and Nkongsamba

^b^Rural sites (forest and humid savanna area) include Mbebe, Nsimalen, Mbandjock, Akonolinga, Campo, Djoum, Soa, Tiko, Loum Mengong, Idenau, Ipono, Dabadi and Manoka

^c^Urban sites (Dry savanna area) include Garoua, Ngaoundéré, Kousseri and Kaélé

^d^Rural sites (Savanna area) include Pitoa, Gounougou, Gashiga, Maga, Boklé, Lagdo and Tibati

^e^Rural sites (grassfields highland area): Foumbot, Ndop, Mangoum, Santchou and Makoutchietoum

*Abbreviations*: *L1014F kdr* allele from West Africa, *L1014S kdr* allele from East Africa, *−* data not available

Resistance to DDT and pyrethroids in *An. gambiae* (*s*.*l*.) was mainly associated with the presence of *kdr* resistant alleles and the overexpression of detoxification genes such as *cyp6m2, cyp6z3* and *cyp6p3* (Table 5). Resistance to the carbamate bendiocarb was attributed to overexpression of several P450 genes. The ACE-1-R mutation conferring resistance to carbamates and organophosphates was not detected in *An. gambiae* populations. In *An. funestus*, resistance mechanisms detected so far in Cameroon include the 119F-GSTe2 mutation conferring resistance to DDT and the 296S-RDL mutation conferring resistance to dieldrin. The implication of P450 monooxygenase enzymes in the resistance to DDT and pyrethroids is suspected.

**Table 5:** Target site resistance and detoxification mechanisms detected for *An. gambiae* and *An. funestus* populations

Species	Insecticide family	Insecticides	Resistance mechanisms		
			Target site	Metabolic detoxification	Reference
*An. gambiae*, *An. coluzzii*	Organochlorines	DDT	L1014F & L1014S	*cyp6m2*, *cyp6p3*, *cyp6p4*, *cyp6z3*, *cyp9k1*, *gstd1-6*, *gstd1-4*	[15, 16, 28, 50]
		Dieldrin	RDL^R^		[3, 73, 74]
	Carbamates	Bendiocarb		*cyp6z3*, *cyp6z1*, *cyp12f2*, *cyp6m3*, *cyp6p4*	[52]
	Pyrethroids	Permethrin & deltamethrin	L1014F & L1014S	*cyp6m2*, *cyp6p3*, *cyp6p4*, *cyp6z3*, *cyp9k1*, *gstd1-6*, *gstd1-4*	[15, 16, 28, 30, 50]
*An. arabiensis*	Organochlorines	DDT	L1014F		[29]
	Pyrethroids	Permethrin & deltamethrin	L1014F	*cyp4g16*, *sod3b*, *gst1-2*, *sod3a*, *sod2*, *tpx4*, *cyp4h24*, *cyp6p3*, *cyp325c2*, *cyp6ag1*	[12, 30, 51]
*An. funestus*	Organochlorines	DDT	119F-GSTe2	Overexpression of P450 Monooxygenase (suspected)	[3, 14]
		Dieldrin	296S-RDL		[3, 14]
	Carbamates	Bendiocarb			
	Pyrethroids	Permethrin & deltamethrin		Overexpression of P450 Monooxygenase (suspected)	[14]

## Discussion

The objective of this review was to provide an update on mosquito susceptibility to insecticides and the evolution of insecticide resistance in the main malaria vectors in Cameroon, i.e. *An. gambiae* (*s*.*l*.) and *An. funestus*, from the 1990s to 2017. Resistance to organochlorine, pyrethroids and carbamates and high susceptibility to organophosphates in the majority of sites was detected and supports the need for frequent monitoring of field population susceptibility. DDT, permethrin, deltamethrin and bendiocarb appeared as the most affected compounds. DDT resistance in Cameroon dates back to the first indoor spraying campaigns conducted across the country in the 1950s [32, 33]. It is likely that cross-resistance to DDT and pyrethroids could have driven rapid emergence and expansion of resistance to pyrethroids. Bendiocarb resistance is more recent and could have resulted from the frequent and uncontrolled use of pesticides in agriculture [34]. In Cameroon, despite high diversity of the vectorial system, insecticide resistance affects mainly the major vector species *An. gambiae* (*s*.*l*.) and *An. funestus* [12, 14, 35]. The fact that these species are by far more affected than any other could have resulted from their high endophagic, endophilic and anthropophilic behaviour, which could expose them more to selective pressure induced by insecticides used for vector control in households. Different resistance evolution patterns were recorded across the country and could point to the probable influence of several factors including historical and contemporary factors shaping resistance in mosquito populations.

### Historical factors

In favour of the global plan for malaria eradication conducted in Africa in the 1950s, pilot programmes for controlling malaria transmission using indoor spraying interventions were implemented in Cameroon [32, 33, 36]. Selected sites for pilot interventions included the area of Yaoundé situated in south Cameroon forest region encompassing Yaoundé and its surroundings, and the area of Maroua and its surroundings in northern Cameroon situated in dry savanna area between the 10 and 12 degrees north latitude [36, 37]. In the forest region, the programme was launched in 1953. Between 1953 and 1956 preliminary trials were conducted using DDT, dieldrin and hexachlorocyclohexane (HCH) [36]. In 1956, the main spraying operations were started in the area of Yaoundé, which was divided into two: the eastern section was treated with diedrin and the western section treated with DDT. HCH, which displayed low residual effect during preliminary tests, was not used [36]. Although a high reduction in mosquito density and plasmodic index (parasite carriage rate) was recorded, the emergence and rapid expansion of dieldrin resistance, which conveyed cross-resistance to hexachlorocyclohexane (HCH) [37], hampered the programme which was eventually stopped in 1960 [36]. In the northern part of the country, spraying with DDT started in 1959. However, this program did not significantly reduce anopheline densities or malaria transmission intensity and plasmodic index [33] while the emergence and spread of DDT resistance was reported [33]. This led to the stoppage of the programme in 1961 [33, 36]. Factors that could have limited the performance of these programmes include financial constraints, change in the biting and resting behaviours of the main vector species, poor implementation, emergence and expansion of dieldrin and DDT resistance in mosquito populations, the limited residual effect of DDT and dieldrin sprayings requiring frequent retreatments [33, 36–38]. The short residual effect of DDT on house construction materials inducing little direct killing of mosquitoes was also reported as a major limitation of DDT spraying programmes in West Africa [39, 40]. Insecticide-based vector control interventions were only reintroduced in the late 1980s following the implementation of pyrethroid-treated net trials across the country [36, 41]. Several pieces of evidence support the fact that the current distribution of DDT and pyrethroid resistance in most parts of Cameroon could have resulted from the intensive use of organochlorines during the indoor spraying programmes of the 1950s. In areas such as Lagdo and Gounougou where indoor spraying campaigns were not undertaken in the 1950s, mosquitoes still display high susceptibility to DDT despite increase resistance to pyrethroids [29, 42]. In contrast, in those areas sprayed, a rapid decrease in mosquito susceptibility to DDT was recorded a few years after the reintroduction of pyrethroid-treated nets [29, 43]. Conversely, it is considered that the absence of any large-scale vector control intervention across the country and limited usage of pesticides in small-scale farming between the 1960s and the 1980s could have favour restoration of full susceptibility of mosquito populations to organochlorines. Indeed, a high susceptibility levels to DDT was detected in sites such as Yaoundé during the first field monitoring programmes conducted in 1997 [44]. Resistance to DDT emerged quickly despite the low usage rate of pyrethroid-treated bednets by the population a few years later [34, 43]. It is likely that after the stoppage of indoor spraying campaigns, frequent use of individual protective measures in households such as spray or coils could have maintained resistant allele presence at a very low frequency in natural populations [27]. From 1990 to 2017, Cameroon also experienced important changes in relation to its demography, urbanization, use of insecticide tools in public health and agricultural practices that could have equally affected the emergence and maintenance of insecticide resistance in vector populations.

### Practice of agriculture

Because the same insecticide classes are used in both public health and agriculture, the largely uncontrolled use of pesticides in agriculture could have affected the efficacy of control tools used in public health. The following has been addressed by several studies conducted across the continent [45–47]. In Cameroon, the size of land used for the practice of small-scale farming in and around major urban centres and in rural settings registered a significant increase during the last few decades following the economic crisis that affected the country [48]. A variety of compounds containing either carbofuran, methyl-parathion, dimethoate, diazinon, endosulfan, cypermethrin, deltamethrin, lambdacyhalothrin, fipronil, chlorpyrifos-ethyl, lambdacyhalothrin were reported used for cabbage, celery, pepper, tomato green bean, watermelon farming [16, 29]. Studies conducted in Douala and Yaoundé, reported a high prevalence of insecticide resistance in mosquitoes originating from agricultural-cultivated sites compared to other sites [15]. High resistance levels to DDT, pyrethroids and carbamates across the country were also reported from cotton, rice or tomatoes growing areas [12, 28, 34, 49]. A contrasting pattern of resistance evolution was registered across the country with the rapid emergence of insecticide resistance in the western highlands of Cameroon where intensive agriculture is practised [16, 29] and slow evolution of insecticide resistance in other sites where agriculture is not practised. The following could likely point to a high selective pressure of pesticides used in agriculture and the limited influence of gene flow in shaping insecticide resistance dissemination across the country. The limited influence of gene flow could probably results from the existence of barriers to gene flow such as the equatorial forest and high mountainous areas yet this still deserves further investigations. In addition to the *kdr* alleles which were largely prevalent in mosquitoes originating from agricultural-cultivated sites, overexpression of several detoxification genes was also reported in mosquito populations [35, 50–52]. Detoxification genes such as *cyp6m2*, *cyp6p3*, *cyp6z3*, *gstd1-6*, emerged as the main compounds driving resistance to DDT and pyrethroids in agricultural-cultivated sites in the city of Yaoundé [50]. Several P450 monooxygenase genes were also reported overexpressed in bendiocarb-resistant mosquitoes deriving from cultivated sites [52]. The profile of genes overexpressed in agricultural sites was also found to vary according to the frequency of pesticide usage. In the city of Lagdo where cotton farms were sprayed with pesticides containing carbamates or cocktails of pyrethroids and organophosphates, studies conducted using the detox chip, reported a different pattern of detoxification genes overexpressed before and during the spraying programme. Before sprayings the following genes were recorded overexpressed, *cyp4h24, cyp6p3, cyp325c2* and *cyp6ag1* whereas the following, *cyp4g16, sod3b, gsts1-2, sod3a, sod2* and *tpx4* were recorded overexpressed during the spraying programme [51].

### Impact of urban pollution

High levels of resistance in mosquito populations were recorded in urban settings and could point to the influence of pollution affecting mosquito population susceptibility to insecticides. Exposition to xenobiotics is known to increase the metabolic capacities of detoxification enzymes in mosquitoes and to confer resistance to insecticides [46, 53]. As a result of poor management of the environment, pollutants generated by industrial or domestic activities can accumulate in the environment affecting mosquito distribution and susceptibility levels to insecticides. Studies assessing the distribution of *An. gambiae* and *An. coluzzii* in the city of Yaoundé indicated that *An. coluzzii* is mainly distributed in the urban environment because it is more tolerant to organic pollutants such as ammonia whereas *An. gambiae* (*s*.*l*.) less tolerant to organic pollutants, is mainly prevalent in rural and periurban areas [19, 54, 55]. Pollutants commonly found in urban breeding sites include hydrocarbons, heavy metals (e.g. Pb, cadmium), organic pollutants (e.g. nitrate, phosphate, NH4+) and polycyclic aromatic hydrocarbons [46, 56]. Further analysis from the cities of Douala and Yaoundé showed that mosquitoes deriving from polluted habitats were more tolerant to insecticides than those collected in unpolluted sites [57]. Laboratory analysis conducted with field population of *An. coluzzii* suggested that exposure of mosquitoes during the larval stage to pollutants such as soap, or hydrogen peroxide reduced the susceptibility level of mosquitoes at the adult stage [58]. Similar observations were also reported for *Ae. albopictus* exposed during the larval stage to benzothiazole resulting in increased tolerance at the adult stage to several insecticides with several detoxification genes overexpressed [46, 59, 60]. In Yaoundé, the analysis of the profile of genes overexpressed in mosquito populations originating from polluted sites showed the overexpression of several detoxification genes such as P450 monooxygenases, glutathione-S transferase and esterases [35, 50]. Although these studies strongly suggest the major influence of pollution driving mosquito tolerance to insecticides, the extent of this selection across the country is not well understood and this deserves further investigation.

### Use of insecticide tools in public health

Since the 1990s, three mass distribution campaigns of treated bed nets have been conducted across Cameroon. The first launched in 2003 concerned mainly children under five years of age and pregnant women and saw the distribution of about two million treated bed nets to the population [7]. The second programme took place in 2011 and concerned the whole country, with over eight million LLINs distributed to the population [7]. The third campaign was conducted in 2014 and consisted of the distribution of more than 12 million LLINs countrywide [61]. It is now estimated that over 60% of the population own nets and 75% use nets regularly [9]. The frequent use of impregnated bed nets alongside spray and coils for fighting against mosquito burden and malaria transmission could induce high selective pressure in mosquito populations. New practices including the use of fumigation by private companies in major urban settings (Yaoundé and Douala) to fight *Culex* or *Aedes* mosquito burdens could have affected the level of susceptibility of mosquitoes to insecticides in recent years. These practices deserve further investigations.

## Conclusion

This review provides an update of insecticide resistance in malaria vector populations in Cameroon and provides a base for discussing further strategies to improve control of field mosquito populations. High insecticide resistance affecting almost all insecticide families used in public health was detected in *An. gambiae* (*s*.*l*.) and *An. funestus*. This stresses the need for further actions to improve control of malaria in the coming years. WHO recommends the use of integrated vector control strategies to improve malaria control and elimination. These strategies would not be efficient unless a clearer picture of the performance of current vector control interventions is available. Reviews like this one could constitute necessary steps to improve strategies to control malaria in the coming years better.

## Abbreviations

DDT: Dichlorodiphenyltrichloroethane
GST: Glutathione-S transferase
HCH: Hexachlorocyclohexane
IRS: Indoor residual spraying
LLINs: Long-lasting insecticidal nets
WHO: World Health Organization

## References

[1] WHO (2015). WHO global malaria programme, world malaria report.

[2] Ranson H, N’Guessan R, Lines J, Moiroux N, Nkuni Z (2011). Pyrethroid resistance in African anopheline mosquitoes: what are the implications for malaria control?. Trends Parasitol.

[3] Wondji CS, Dabire RK, Tukur Z, Irving H, Djouaka R, Morgan JC (2011). Identification and distribution of a GABA receptor mutation conferring dieldrin resistance in the malaria vector *Anopheles funestus* in Africa. Insect Biochem Mol Biol.

[4] Wondji C, Irving H, Morgan J, Lobo N, Collins F, Hunt R (2009). Two duplicated P450 genes are associated with pyrethroid resistance in *Anopheles funestus*, a major malaria vector. Genome Res.

[5] Riveron J, Irving H, Ndula M, Barnes K, Ibrahim S, Paine M (2013). Directionally selected cytochrome P450 alleles are driving the spread of pyrethroid resistance in the major malaria vector *Anopheles funestus*. Proc Natl Acad Sci USA.

[6] Programme WGM. Global plan for insecticide resistance management in malaria vectors (GPIRM). Geneva: World Health Organization; 2012.

[7] PNLP (2012). Plan Strategique nationale de lutte contre le paludisme 2011–2015. Rapport Minsante Cameroun.

[8] Etang J, Fondjo E, Bintsindou P, Bagayoko M, Manga L (2007). Profil entomologique du Cameroun. Rapport Minsanté Cameroun et OMS.

[9] Bowen H (2013). Impact of a mass media campaign on bed net use in Cameroon. Malar J.

[10] https://lcclc.info/index.php/2017/04/19/cameroon-health-about-2600-deaths-caused-by-malaria-in-2016: Accessed 18 Apr 2017.

[11] Etang J, Pennetier C, Piameu M, Bouraima A, Chandre F, Awono-Ambene P (2016). When intensity of deltamethrin resistance in *Anopheles gambiae* (*s.l.*) leads to loss of long lasting insecticidal nets bio-efficacy: a case study in north Cameroon. Parasit Vectors.

[12] Chouaibou M, Etang J, Brevault T, Nwane P, Hinzoumbe C, Mimpfoundi R (2008). Dynamics of insecticide resistance in the malaria vector *Anopheles gambiae s.l.* from an area of extensive cotton cultivation in northern Cameroon. Tropical Med Int Health.

[13] Antonio-Nkondjio C, Tene Fossog B, Kopya E, Poumachu Y, Menze-Djantio B, Ndo C (2015). Rapid evolution of pyrethroid resistance prevalence in *Anopheles gambiae* populations from the cities of Douala and Yaoundé (Cameroon). Malar J.

[14] Menze BD, Riveron JM, Ibrahim SS, Irving H, Antonio-Nkondjio C, Awono-Ambene PH (2016). Multiple insecticide resistance in the malaria vector *Anopheles funestus* from northern Cameroon is mediated by metabolic resistance alongside potential target site insensitivity mutations. PLoS One.

[15] Antonio-Nkondjio C, Fossog B, Ndo C, Djantio B, Togouet S, Awono-Ambene P (2011). *Anopheles gambiae* distribution and insecticide resistance in the cities of Douala and Yaounde (Cameroon): influence of urban agriculture and pollution. Malar J.

[16] Nwane P, Etang J, Chouaibou M, Toto J, Kerah-Hinzoumbe C, Mimpfoundi R (2009). Trends in DDT and pyrethroid resistance in *Anopheles gambiae s.s.* Populations from urban and agro-industrial settings in southern Cameroon. BMC Infect Dis.

[17] WHO (2013). Test procedures for insecticide resistance monitoring in malaria vector mosquitoes.

[18] Simard F, Ayala D, Kamdem G, Pombi M, Etouna J, Ose K (2009). Ecological niche partitioning between *Anopheles gambiae* molecular forms in Cameroon: the ecological side of speciation. BMC Ecol.

[19] Kamdem C, Fossog B, Simard F, Etouna J, Ndo C, Kengne P (2012). Anthropogenic habitat disturbance and ecological divergence between incipient species of the malaria mosquito *Anopheles gambiae*. PLoS One.

[20] Wondji C, Frederic S, Petrarca V, Etang J, Santolamazza F, Della Torre A (2005). Species and populations of the *Anopheles gambiae* Complex in Cameroon with special emphasis on chromosomal and molecular forms of *Anopheles gambiae s.s*. J Med Entomol.

[21] Antonio-Nkondjio C, Kerah C, Simard F, Awono-Ambene H, Mouhamadou C, Tchuinkam T (2006). Complexity of malaria vectorial system in Cameroon: contribution of secondary vectors to malaria transmission. J Med Entomol.

[22] Ayala D, Carlo C, Ose K, Kamdem G, Antonio-Nkondjio C, Agbor J (2009). Habitat suitability and ecological niche profile of major malaria vectors in Cameroon. Malar J.

[23] Le Goff G, Robert V, Carnevale P (1994). Evaluation d'un répulsif à base de DEET sur trois vecteurs du paludisme en Afrique centrale. Cah Sante AUPELF UREF.

[24] Le Goff G, Robert V, Fondjo E, Carnevale P (1992). Efficacy of insecticide impregnated bednets to control malaria in a rural forested area in southern Cameroon. Mem Inst Oswaldo Cruz.

[25] Manga L, Robert V, Carnevale P (1995). Efficacité des serpentin et des diffuseurs en plaque dans la protection contre les vecteurs du paludisme au Cameroun. Cah Sante AUPELF UREF..

[26] Antonio-Nkondjio C, Demanou M, Etang J, Bouchite B (2013). Impact of cyfluthrin (Solfac EW050) impregnated bed nets on malaria transmission in the city of Mbandjock: lessons for the nationwide distribution of long-lasting insecticidal nets (LLINs) in Cameroon. Parasit Vectors.

[27] Desfontaines M, Gelas H, Cabon H, Goghomu A, Kouka-Bemba D, Carnevale P, Evaluation d (1990). pratiques et des coûts de lutte antivectorielle à l'échelon familial en Afrique Centrale. II Ville de Douala (Cameroun). Ann Soc Belg Med Trop.

[28] Etang J, Fondjo E, Chandre F, Brengues C, Nwane P, Chouaubou M (2006). First report of knockdown mutations in the malaria vector *Anopheles gambiae* from Cameroon. Am J Trop Med Hyg.

[29] Ndjemai H, Patchoke S, Atangana J, Etang J, Simard F, Bilong Bilong C (2008). The distribution of insecticide resistance in *Anopheles gambiae s.l.* populations from Cameroon: an update. Trans R Soc Trop Med Hyg.

[30] http://www.malariaeradication.org/knowledge-hub/astmh-2016-jude-d-bigoga-implications-insecticide-resistance-assessing-impact: Accessed 16 Nov 2016.

[31] Knox T, Juma E, Ochomo E, Pates Jamet H, Ndungo L, Chege P (2014). An online tool for mapping insecticide resistance in major *Anopheles* vectors of human malaria parasites and review of resistance status for the Afrotropical region. Parasit Vectors.

[32] Mouchet J, Cavalie P, Callies J, Marticou HL (1961). irritabilite vis a vis du DDT d' *Anopheles gambiae* et d'*A. funestus* dans le Nord-Cameroun. Riv Malariol.

[33] Cavalie P, Mouchet J (1962). Les campagnes experimentales d'eradication du paludisme dans le Nord de la Republique du Cameroun. Med Trop.

[34] Bigoga JD, Manga L, Titanji VPK, Etang J, Coetzee M, Leke RGF (2007). Susceptibility of *Anopheles gambiae* Giles (Diptera: Culicidae) to pyrethroids, DDT and carbosulfan in coastal Cameroon. Afr Entomol.

[35] Nwane P, Etang J, Chouaibou M, Toto J, Koffi A, Mimpfoundi R (2013). Multiple insecticide resistance mechanisms in *Anopheles gambiae s.l.* populations from Cameroon, Central Africa. Parasit Vectors.

[36] Carnevale P, Mouchet J (2001). La lutte antivectorielle au Cameroun. Passé-Présent-Avenir. Réflexions. Bull Soc Pathol Exot.

[37] Livadas G, Mouchet J, Gariou J, Chastang R (1958). Peut-on envisager l'eradication du paludisme dans la region forestiere du Sud-Cameroun?. Rev Malariol.

[38] Gariou J, Mouchet J (1961). Apparition d'une souche d'*Anopheles gambiae* resistante a la dieldrine dans la zone de campagne antipaludique du Sud-Cameroun. Bull Soc Path Exot.

[39] Hamon J, Mouchet J, Brengues J, Chauvet G (1970). Problems facing Anopheline vector control. Vector ecology and behaviour before, during, and after application of control measures. Misc Publ Entomol Soc Am.

[40] Snowden F, Bucala R (2014). The global challenge of malaria: pasts lessons and future prospects.

[41] Carnevale P, Bitsindou P, Diomande L, Robert V (1992). Insecticide impregnation can restore the efficiency of torn bednets and reduce man-vector contact in malaria endemic areas. Trans R Soc Trop Med Hyg.

[42] Antonio-Nkondjio C, Atangana J, Ndo C, Awono-Ambene P, Fondjo E, Fontenille D (2008). Malaria transmission and rice cultivation in Lagdo, northern Cameroon. Trans R Soc Trop Med Hyg.

[43] Etang J, Manga L, Chandre F, Guillet P, Fondjo E, Mimpfoundi R (2003). Insecticide susceptibility status of *Anopheles gambiae s.l.* (Diptera: Culicidae) in the Republic of Cameroon. J Med Entomol.

[44] Chandre F, Darrier F, Manga L, Akogbeto M, Faye O, Mouchet J (1999). Status of pyrethroid resistance in *Anopheles gambiae sensu lato*. Bull World Health Organ.

[45] Yadouleton A, Asidi A, Djouaka R, Braima J, Agossou C, Akogbeto M (2009). Development of vegetable farming: a cause of the emergence of insecticide resistance in populations of *Anopheles gambiae* in urban areas of Benin. Malar J.

[46] Nkya T, Akhouayri I, Kisinza W, David J (2013). Impact of environment on mosquito response to pyrethroid insecticides: facts, evidences and prospects. Insect Biochem Mol Biol.

[47] Chouaïbou MS, Fodjo BK, Fokou G, Koudou BG, David J-P, Allassane OF (2016). Influence of the agrochemicals used for rice and vegetable cultivation on insecticide resistance in malaria vectors in southern Côte d’Ivoire. Malar J.

[48] Yengoh GT, Ardö J (2014). Crop yield gaps in Cameroon. Ambio.

[49] Barnes KG, Weedall GD, Ndula M, Irving H, Mzihalowa T, Hemingway J, et al. Genomic footprints of selective sweeps from metabolic resistance to pyrethroids in African malaria vectors are driven by scale up of insecticide-based vector control. PLOS Genet. 2017;13:e1006539. 10.1371/journal.pgen.1006539 PMC5289422 28151952

[50] Fossog Tene B, Poupardin R, Costantini C, Awono-Ambene P, Wondji CS, Ranson H (2013). Resistance to DDT in an urban setting: common mechanisms implicated in both M and S forms of *Anopheles gambiae* in the City of Yaoundé, Cameroon. PLoS One.

[51] Muller P, Chouaibou M, Pignatelli P, Etang J, Walker E, Donnelly M (2007). Pyrethroid tolerance is associated with elevated expression of antioxidants and agricultural practice in *Anopheles arabiensis* sampled from an area of cotton fields in northern Cameroon. Mol Ecol.

[52] Antonio-Nkondjio C, Poupardin R, Tene BF, Kopya E, Costantini C, Awono-Ambene P (2016). Investigation of mechanisms of bendiocarb resistance in *Anopheles gambiae* populations from the city of Yaoundé, Cameroon. Malar J.

[53] Li X, Schuler M, Berenbaum M (2007). Molecular mechanisms of metabolic resistance to synthetic and natural xenobiotics. Annu Rev Entomol.

[54] Tene Fossog B, Ayala D, Acevedo P, Kengne P, Ngomo Abeso Mebuy I, Makanga B (2015). habitat segregation and ecological character displacement in cryptic African malaria mosquitoes. Evol Appl.

[55] Tene Fossog B, Antonio-Nkondjio C, Kengne P, Njiokou F, Besansky N, Costantini C (2013). Physiological correlates of ecological divergence along an urbanization gradient: differential tolerance to ammonia among molecular forms of the malaria mosquito *Anopheles gambiae*. BMC Ecol.

[56] Mireji P, Keating J, Hassanali A, Mbogo C, Nyambaka H, Kahindi S (2008). Heavy metals in mosquito larval habitats in urban Kisumu and Malindi, Kenya, and their impact. Ecotoxicol Environ Saf.

[57] Tene Fossog B, Kopya E, Ndo C, Menze-Djantio B, Costantini C, Njiokou F (2012). Water quality and *Anopheles gambiae* larval tolerance to pyrethroids in the cities of Douala and Yaounde (Cameroon). J Trop Med.

[58] Antonio-Nkondjio C, Youmsi-Goupeyou M, Kopya E, Tene Fossog B, Njiokou F, Costantini C (2014). Exposure to disinfectants (soap and hydrogen peroxide) increases tolerance to permethrin in *Anopheles gambiae* populations from the city of Yaoundé, Cameroon. Malar J.

[59] Poupardin R, Reynaud S, Strode C, Ranson H, Vontas J, David J (2008). Cross-induction of detoxification genes by environmental xenobiotics and insecticides in the mosquito *Aedes aegypti*: impact on larval tolerance to chemical insecticides. Insect Biochem Mol Biol.

[60] Nkya T, Akhouayri I, Poupardin R, Batengana B, Mosha F, Magesa S (2014). Insecticide resistance mechanisms associated with different environments in the malaria vector *Anopheles gambiae*: a case study in Tanzania.. Malar J.

[61] Gregoire J, Oboussoumi E, Phollet E. Alliance for malaria prevention Cameroon mission report march 24th to April 18th, 2015. Report alliance for Malaria Prevention; 2015:1–45.

[62] Antonio-Nkondjio C, Defo-Talom B, Tagne-Fotso R, Tene-Fossog B, Ndo C, Lehman L (2012). High mosquito burden and malaria transmission in a district of the city of Douala, Cameroon. BMC Infect Dis.

[63] Bigoga J, Ndangoh D, Awono-Ambene P, Patchoke S, Fondjo E, Leke R (2012). Pyrethroid resistance in *Anopheles gambiae* from the rubber cultivated area of Niete, south region of Cameroon. Acta Trop.

[64] Etang J, Manga L, Toto J, Guillet P, Fondjo E, Chandre F (2007). Spectrum of metabolic-based resistance to DDT and pyrethroids in *Anopheles gambiae s.l* populations from Cameroon. J Vect Ecol.

[65] Etang J, Vicente J, Nwane P, Chouaibou M, Morlais I, Do Rosario V (2009). Polymorphism of intron-1 in the voltage-gated sodium channel gene of *Anopheles gambiae* ss populations from Cameroon with emphasis on insecticide knockdown resistance mutations. Mol Ecol.

[66] Etang J, Nwane P, Mbida J, Piameu M, Manga B, Souop D (2011). Variations of insecticide residual bio-efficacy on different types of walls: results from a community-based trial in south Cameroon. Malar J.

[67] Ntonga Akono P, Tonga C, Mbida Mbida J, Ngo Hondt O, Awono-Ambene H, Ndo C (2015). *Anopheles gambiae*, vecteur majeur du paludisme à Logbessou, zone péri-urbaine de Douala (Cameroun). Bull Soc Pathol Exot.

[68] Ntonga Akono P, Mbouangoro A, Mbida Mbida J, Ndo C, Peka Nsangou M, Kekeunou S (2017). Le complexe d'espèces *Anopheles gambiae* et le gène de résistance kdr en périphérie de Douala. Cameroun Bull Soc Path Exot.

[69] Nwane P, Etang J, Chouaibou M, Toto J, Mimpfoundi R, Simard F (2011). Kdr-based insecticide resistance in *Anopheles gambiae s.s.* populations in Cameroon: spread of the L1014F and L1014S mutations. BMC Res Notes.

[70] Reimer L, Fondjo E, Patchoke S, Diallo B, Lee Y, Arash N (2008). Relationship between kdr mutation and resistance to pyrethroid and DDT insecticides in natural populations of *Anopheles gambiae*. J Med Entomol.

[71] Santolamazza F, Calzetta M, Etang J (2008). Distribution of knock-down resistance mutations in *Anopheles gambiae* molecular forms in west and west-central Africa. Malar J.

[72] Moyou-Somo R, Lehman L, Awahmukalah S, Ayuk Enyong P (1995). Deltamethrin impregnated bednets for the control of urban malaria in Kumba town, South-West Province of Cameroon. J Trop Med Hyg.

[73] Corbel V, N'Guessan R, Brengues C, Chandre F, Djogbenou L, Martin T (2007). Multiple insecticide resistance mechanisms in *Anopheles gambiae* and *Culex quinquefasciatus* from Benin, West Africa. Acta Trop.

[74] Kwiatkowska R, Platt N, Poupardin R, Irving H, Dabire R, Mitchell S (2013). Dissecting the mechanisms responsible for the multiple insecticide resistance phenotype in *Anopheles gambiae* s.s., M form, from Vallee du Kou, Burkina Faso. Gene.

